#  C677T (RS1801133 ) MTHFR gene polymorphism frequency in a colombian population 

**Published:** 2015-06-30

**Authors:** Consuelo Romero-Sánchez, Alberto Gómez-Gutierrez, Piedad Elena Gómez, Maria Consuelo Casas-Gomez, Ignacio Briceño

**Affiliations:** 1 Instituto de Referencia Andino. Bogotá, Colombia; 2 Instituto de Genética Humana, Facultad de Medicina, Pontificia Universidad Javeriana. Bogotá, Colombia; 3 Facultad de Medicina, Universidad de La Sabana. Bogotá, Colombia; 4 Servicio de Reumatología e Inmunología, Hospital Militar. Bogotá, Colombia; 5 Instituto UIBO - Unidad de Investigación Básico Oral, Universidad El Bosque. Bogotá, Colombia

**Keywords:** Methotrexate, genotype, alleles, polymorphism, metylentetrahidrofolate-reductase

## Abstract

**Introduction:**

Abnormal levels of the enzyme methylenetetrahydrofolate reductase (MTHFR) are associated with an increased risk of both cardiovascular and cerebrovascular disease and higher concentrations of homocysteine. Abnormal levels are also related to birth defects, pregnancy complications, cancer and toxicity to methotrexate (MTX). Polymorphisms of *MTHFR *affect the activity of the enzyme. Genetic associations have been related to treatment efficacy.

**Objective:**

To establish the frequency of the C> T polymorphism at nucleotide 677 of the MTHFR gene in a group of Colombian individuals.

**Methods:**

Data from pharmacogenetic microarrays that include MTX sensibility-associated polymorphisms were retrospectively collected (Pathway Genomics^®)^. The frequency of the C> T MTHFR rs1801133 marker polymorphism was analyzed.

**Results:**

Microarray data from 68 men and 84 women were analyzed. Comparisons of genotype C/C vs. C/T and T/T were statistically significantly different (*p*= 0.00, *p*= 0.026, respectively), as were C/T and T / T (*p*= 0.0001).

**Conclusions:**

Results for the C/C and C/T genotypes in a Colombian population are similar to other previously studied groups of healthy subjects. Subjects from our population might be at risk of developing diseases associated with *MTHFR* polymorphisms and might present toxicity and adverse effects if treated with MTX, which suggests the need to evaluate therapeutic alternatives based on individual pharmacogenetic studies.

## Abstract (es)

**Introducción:**

Las alteraciones de la enzima metilen-tetrahidrofolato reductasa (MTHFR) se asocian con riesgo cardiovascular y cerebrovascular y con presencia de concentraciones altas de homocisteína. Se relacionan también con defectos congénitos, complicaciones en embarazo, cáncer y toxicidad del Metotrexato (MTX). Los polimorfismos del gen MTHFR afectan la actividad de la enzima. Se han descrito asociaciones genéticas con la eficacia del tratamiento con MTX.

**Objetivo:**

Establecer la frecuencia del polimorfismo C>T en el nucleótido 677 del gen MTHFR en un grupo de individuos Colombianos.

**Métodos:**

Estudio descriptivo de corte transversal. Se recolectaron retrospectivamente resultados de microarreglos farmacogenéticos que incluyen polimorfismos asociados con la sensibilidad al MTX (PathwayGenomics(r)). Se analizó la frecuencia del polimorfismo C>T del polimorfismo rs1801133 del gen MTHFR.

**Resultados:**

Se analizaron microarreglos de 68 hombres y 84 mujeres. Las comparaciones del genotipo C/C frente a C/T y a T/T fueron estadísticamente significativas (*p*= 0.001 y *p*= 0.026 respectivamente) tanto como la comparación entre C/T y T/T (*p*= 0.0001).

**Conclusiones:**

Los genotipos C/C y C/T en Colombia son tan variables como en otros grupos sanos en otras poblaciones. Los sujetos de nuestra población podrían tener riesgo para el desarrollo de enfermedades asociadas al polimorfismo del genMTHFR y con genotipos de riesgo de presentar toxicidad y efectos adversos del MTX, lo cual sugiere la necesidad de evaluar alternativas terapéuticas con estudios farmacogenéticos.

## Introduction

 The sequencing of the human genome and the study of single nucleotide polymorphisms (SNPs) have generated a considerable amount of data that reveal the basis of the genetic association of hundreds of diseases and toxicity, as well as the efficacy of various drugs. The latter area of study is called "pharmacogenetics". The technology applied to reveal these associations is available, and costs are becoming progressively reduced in relation to the benefit of optimization and clinical application of their findings. 

 The response of each individual to the same treatment can be in part, to heritable factors, creating differences in drug efficacy among subjects due to polymorphisms in genes that encode enzymes involved in the metabolism of certain drugs, in their transporters and/or specific treatment targets 1. 

 A gene is described as "polymorphic" when allelic variants are present. The activity of the protein can vary when compared to the original allele. Pharmacogenetics may additionally explain individual differences in response to treatment and may have predictive value for patient response to various drugs 2,3. 

 A classic example of pharmacogenetics is rheumatoid arthritis (RA), an autoimmune disease that affects between 0.5% and 1% of the population. One of the most widely used therapies in this disease, which is one of the most effective from the clinical standpoint, is methotrexate (MTX). Several reports describe its use since 1951, and clinical trials show its effectiveness but also demonstrate its toxicity, including increased nodes, pneumonitis, neurologic involvement, gastrointestinal complications such as nausea, vomiting and diarrhea, increased transaminases, hematologic abnormalities, skin rash, stomatitis and alopecia 4-6. Genetic variants of the *MTHFR* gene related to the efficacy of MTX have been studied. The C677T polymorphism enzyme activity was reduced by 35% in carriers and has a prevalence of 40% in the population. MTX activity is reduced 50-70% in homozygous TT at position 677 with a prevalence of 8-10% in the population 7. 

 Additionally, MTX inhibits dihydrofolate reductase (DHFR) and folate-dependent enzymes such as timidilatosintase (TS) and methylenetetrahydrofolate reductase (MTHFR). It has been shown that their polymorphisms affect enzyme activity, resulting in elevated homocysteine ​​levels and toxicity 8. 

 The study of the MTHFR enzyme has acquired much interest after the fundamental discovery by Kang *et al*. 7, who reported that a variant of the thermolabile enzyme was associated with increased cardiovascular risk and a higher concentration of total plasma homocysteine. Today, the concentration of homocysteine ​​in plasma is considered a risk factor for coronary heart disease, cerebrovascular disease, and vascular occlusion 9. It has also been linked to the occurrence of neural tube defects and other birth defects, complications associated with pregnancy 10,11 and cancer 12. 

 Genetic analysis can be requested for the *MTHFR* gene alone, or in the context of molecular genetic profiles as part of preventive medicine. These profiles evaluate pharmacogenetic associations with drugs, including MTX. The polymorphism analysis can also be performed by direct sequencing of the entire gene or a fragment there of, by analysis using restriction enzyme polymorphisms (RFLP) or by microarray techniques. The spectrum of the frequency of the *MTHFR* gene polymorphism C677T in a group of healthy Colombian individuals was determined to evaluate the genetic background in relation to disease susceptibility and pharmacogenetic applications. 

## Materials and Methods

 A retrospective cross-sectional study in 152 healthy Colombian individuals over 18 yrs was conducted using molecular genetic profiling. DNA samples were collected from saliva samples in the area of ​​preventive medicine of the "Instituto de Referencia Andino", including all applications from medical orders and/or direct individuals received between the years 2012 and 2014. All individuals signed an informed consent. Subjects for whom demographic data were not complete were excluded. 

 The pharmacogenetic polymorphism panel from PathwayGenomics^®^ in *Medication DNA Insight* *TM* *genetic test* evaluates 15 metabolism associations for drug metabolism, including MTX in addition to Abacavir^®^ hypersensitivity, aminoglycoside-induced ototoxicity, response to beta blockers, carbamazepine hypersensitivity, clopidogrel metabolism, estrogen supplementation, alpha interferon, metoprolol metabolism, metabolism and hypersensitivity to phenytoin, proton-pump inhibitor, simvastatin induced myopathy, metabolism of voriconazole and warfarin. 

DNA microarrays were used to measure expression levels of probes, corresponding to allele mutations "C" and "T" in the *MTHFR* gene. Each microarray position contains 10-12 picomoles of a specific DNA sequence under high stringency. Probe-target hybridization was detected and quantified by fluorophores attached to the probe. The frequency of the C677T polymorphism, consisting of a C>T substitution in the *MTHFR* gene marker rs1801133, was analyzed. 

 Each participant was identified by a code used in a database, where the descriptive analysis for continuous variables such as age was performed with measures of central tendency and Shapiro Wilk normality test. The description of categorical variables (origin, sex, genotype and allele) was performed by frequency analysis. Given the variable nature of the study association levels, they were evaluated by a Chi square test (X²) with a confidence level of 95%. In addition, Hardy Weinberg equilibrium was tested. The results were transferred to the SPSS^®^ statistical program for Windows^®^ V20. They were not stratified by ethnic group or socioeconomic levels. 

### Ethical considerations

Study was approved by the Ethics Committee of Fundacion Instituto de Reumatología Fernando Chalem.

## Results

 The study included 152 individuals, 68 men (44.7%) and 84 women (55.3%) aged 40 ±13 and 41 ±13 yrs, respectively. The origins of the samples were as follows: Medellin (15 individuals: 9.9%), Cali (10 individuals: 6.6%), Bogota (119 individuals: 78.3%), Barranquilla and Cartagena (6 subjects: 3.9%), Pasto (2 Individuals: 1.3%).

**Figure 1.**
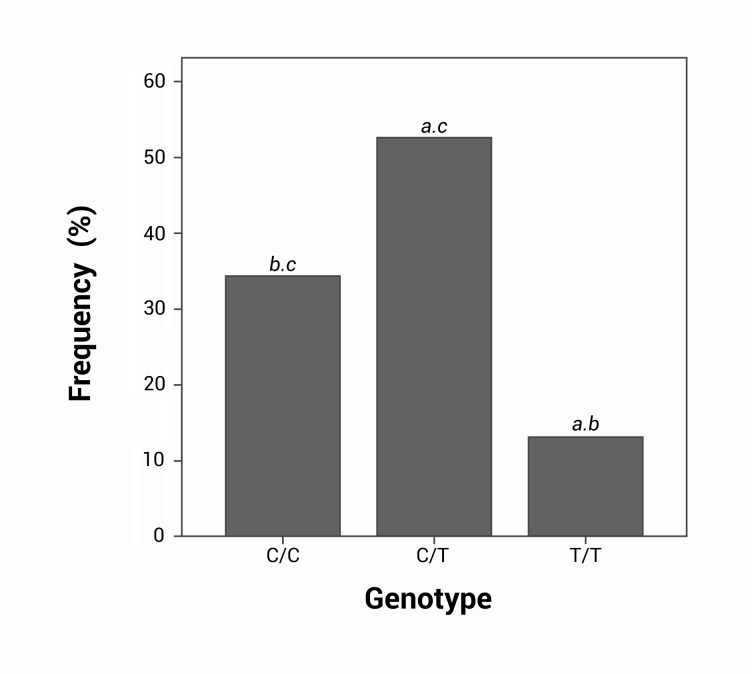
Comparison of the three genotypes in the total sample: **a** significant statistical differences for C/C, **b** significant statistical differences for C/T, **c** significant statistical differences for T/T. All the analysis were obtained by Chi square p< 0.05.

 The most frequently observed genotype was the heterozygous C/T (80 individuals, 52.6%), followed by the homozygous C/C with 52 individuals (34.2%). The homozygous genotype T/T had the lowest frequency in the population studied (20 individuals), corresponding to 13.2%. The frequencies of genotype C/C vs. C/T and T/T were significantly different (*p* = 0.001 and *p*= 0.026, respectively), as were C/T and T/T *p*= 0.0001 (Fig. 1). 

 No statistically significant associations were observed between genotype and allele with sex, *p*= 0.799 and *p*= 0.615, respectively, using a Chi square test (X²) (Table 1). The population is in Hardy-Weinberg equilibrium (*p*= 0.211). 

**Table 1.**
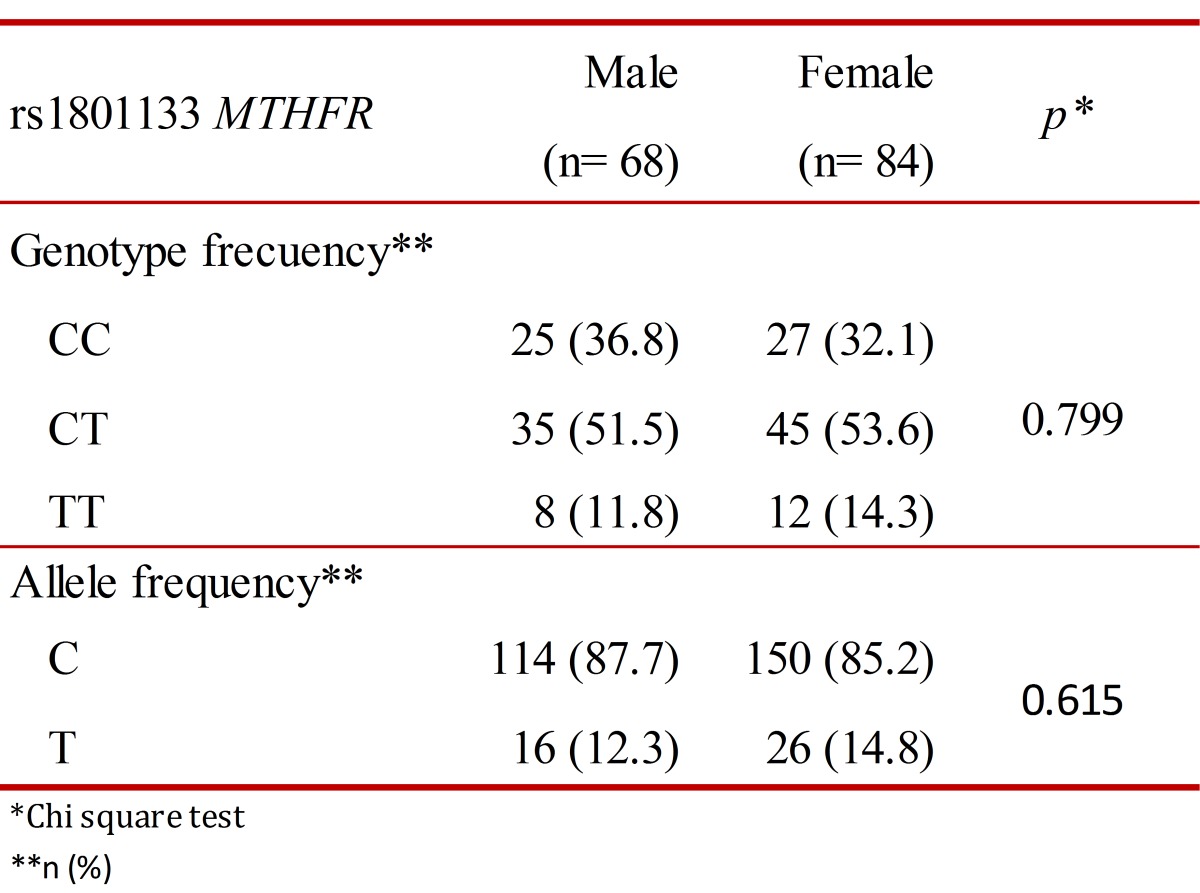
Genotype and allele frequency.

 In assessing the allele frequency in the population, the C allele was found in 86.3% (n= 306) of individuals and the T allele in 13.7% (n= 42). 

## Discussion

 There are functional polymorphisms in genes encoding enzymes that are involved in metabolism that may produce susceptibility to diseases. For that reason, *MTHFR* gene polymorphisms are among the most studied 13-15. 

In the current study, the population was in Hardy-Weinberg equilibrium for the different alleles of the *MTHFR* gene, and therefore, there is no evidence of selection for any of them. Because it has been found that this polymorphism affects MTHFR enzyme activity, it is important to assess its frequency and its effect on the population. 

The MTHFR enzyme is a critical protein in the metabolism of 5-methyl tetrahydrofolate reductase, which is the donor in several biochemical reactions involved in the methylation of homocysteine. A dozen polymorphisms of the *MTHFR* gene have been described, but two main variants from the functional point of view are the most studied: A1298C and C677T. The organic effect of these polymorphisms has been determined, generating predictive results for patient response to MTX 7. The results of a meta-analysis evaluating the C677T polymorphism, homozygous or heterozygous, show great variability between populations 16-19. 

Most genotype frequency observed in the population corresponded to the heterozygous C/T, followed by C/C, with the lowest frequency for the T/T genotype. The C677T polymorphism produces a change to alanine from valine at codon 222, corresponding to a thermolabile MTHFR enzyme variant that generates a decrease in activity and an increased plasma level of homocysteine 20. The homozygous genotype T/T has 30% of the original activity with a prevalence of 8 to 10% in the general population. Heterozygotes have approximately 60% of normal activity and a prevalence of 40% in the population 20. 

A study conducted in Mexico, in which the frequency of *MTHFR* gene polymorphisms in women with and without cervical cancer was evaluated, showed a C/C genotype in 22.4% of the 89 control women with an average age of 44 years old, lower than the 32.1% found in the present study. Moreover, genotype C/T was present in 55.1% of the study population, similar to that found in the group of Colombian women analyzed (53.6%). Finally, the T/T genotype was found in 22.5% of the population, a figure significantly higher than in the present study, considering that this is the homozygous variant that is associated with an increased risk of cancer. Allele frequencies of both the C and T alleles were 0.5 in the Mexican study 21, which contrasts with the population analyzed herein, in which the predominant was the C allele. 

Several previous studies have been conducted in Colombia 22-25, in which the allele and genotype frequencies of this polymorphism were analyzed by comparing cases and controls, always with the aim to determine their association with different pathologies. 

Bermudez *et al*. studied 102 volunteers of both sexes (53 women and 49 men) from various parts of Colombia residing in Bogota, aged between 18 and 50 yrs old 22. This population was not stratified by origin, ethnicity or socioeconomic status. The T/T genotype showed a frequency of 27.4%, higher than the value found in the present study. In women, the frequency was 13.2%, a 14.3% increase. In another evaluation of Colombian individuals, healthy subjects were included, and different genotype frequencies were found relative to the present report: C/C allele in 22.5% and T/T 27.4%, with minor allele frequencies for C (47.5%) and T (52.4%), the allele predominantly associated with toxicity for MTX 23. These reports of healthy Colombian individuals included smaller numbers. They were analyzed with RFLP methodology, unlike this study, in which microarrays were used. 

In a study of a population sample of 206 students in Bogota, Colombia, overall frequencies were calculated using data from healthy controls reported in other studies, finding similar frequencies of genotypes and alleles detected in the group of subjects analyzed in our study 26. That sample was not in Hardy-Weinberg equilibrium, while the overall Colombian data used in the present study was found to be in equilibrium. 

The frequency of TT homozygosity in other populations worldwide varies from 0% to over 30% 26. However, the results of our study are similar to those reported by González et al., in a greater number of healthy Colombian individuals 19. These differences were also found in China 27,28 and the United States 29, where analyses were reported in subjects from the same geographic population, in which very different results were obtained. In the United States, in a study involving different ethnic groups, a lower frequency of the T allele was found in African Americans and a higher frequency was found in a Hispanic population 29, in contrast to our results in which the T allele was found in 13.7% of the population tested. Worldwide frequencies range from 1% in a population from Southeast India 30 to 43% in China 27,31. 

 In summary, substantial variability has been observed in the frequencies of C and T alleles worldwide. In Colombia, some studies show a predominance of allele C and others of allele T. Differences between populations suggest that both allele frequencies in certain circumstances could produce selective advantages and disadvantages. Some reports suggest that the T allele can produce resistance to infections such as malaria and cytomegalovirus and may also protect against hypertension 32,33. Similarly, the C allele protects against birth defects and cancer and is associated with higher fertility and relatively late onset of neurological diseases 34. These findings allow postulating that the frequencies of the C and T alleles may be conditioned locally in different populations by environmental selective pressures in different regions of Colombia and the world. 

 The results of the present study are limited to a Colombian population who applied to study their genetic profile in the cities of Bogota, Medellin, Barranquilla and Cali, which does not afford a comprehensive understanding of the Colombian population. The findings in different studies have shown variation, even when people have been analyzed in the same geographical area. It is possible that the differences found in the different studies may have resulted from different methodological approaches used and the ethnic heterogeneity of the subjects 24,25. 

 Some subjects in our population with *MTHFR* gene polymorphism genotypes associated with toxicity and adverse defects to MTX could be at high risk, suggesting the need to evaluate therapeutic alternatives with pharmacogenetic studies of MTX genotype T/T. From the clinical perspective, the classification of patients for polymorphisms of the MTHFR gene before administering medication would offer alternative treatments without subjecting patients to harmful effects, optimizing therapy and reducing costs for health systems. 

## Conclusion

 The results for the genotypes C/C and C/T in Colombia are as variable as in other groups of healthy individuals studied in other populations, and this variability may be subject to selective pressures of the environment in different regions of Colombia.
